# Lassa fever: the challenges of curtailing a deadly disease

**Published:** 2012-03-23

**Authors:** Titus Ibekwe

**Affiliations:** 1 Department of Surgery(ENT), College of Health Sciences University of Abuja and University of British Columbia(UBC), Vancouver Canada

**Keywords:** Lassa fever, Hemorrhagic illness, sensorineural hearing loss, West African

## Abstract

Today Lassa fever is mainly a disease of the developing world, however several imported cases have been reported in different parts of the world and there are growing concerns of the potentials of Lassa fever Virus as a biological weapon. Yet no tangible solution to this problem has been developed nearly half a decade after its identification. Hence, the paper is aimed at appraising the problems associated with LAF illness; the challenges in curbing the epidemic and recommendations on important focal points. A Review based on the documents from the EFAS conference 2011 and literature search on PubMed, Scopus and Science direct. The retrieval of relevant papers was via the University of British Columbia and University of Toronto Libraries. The two major search engines returned 61 and 920 articles respectively. Out of these, the final 26 articles that met the criteria were selected. Relevant information on epidemiology, burden of management and control were obtained. Prompt and effective containment of the Lassa fever disease in Lassa village four decades ago could have saved the West African sub-region and indeed the entire globe from the devastating effect and threats posed by this illness. That was a hard lesson calling for much more proactive measures towards the eradication of the illness at primary, secondary and tertiary levels of health care.

## To the editors of the Pan African Medical Journal

Today, Lassa fever is mainly a disease of the developing world, however, several imported cases with hazardous outcomes have been reported in different parts of the world including North America [1–3] Europe [4–6] and Asia[7,8] etc. Growing concern of the potentials of Lassa fever virus (LAV) as biological weapon is real, yet no lasting solution to this problem has been developed nearly half a century of its identification in a remote village in Nigeria. To this end, the paper is aimed at appraising the problems associated with LAF illness; the challenges in curbing the epidemic and recommendations on important focal points.

Information were obtained from the proceedings of the African session on “Infectious diseases and hearing loss in Africa” at the European Federation of Audiology Conference (EFAS), Warsaw 2011. The members of the panel of discussants at the conference were experts drafted from different parts of Africa [9]. The other sources of information include literature search at (1) PubMEd, (2) Scopus and Science direct with the mesh words “Lassa fever”, “Epidemiology”, “symptoms” and “control”. The University of British Columbia and Toronto Libraries were also utilized for retrievals of essential articles. Furthermore, resource information was also obtained from the websites of World Health Organization (WHO) and Center for Disease Control and Prevention (CDC). The two major search engines returned 61 and 920 articles respectively. All animal and experimental studies were excluded and only those papers directly focused on the topic were considered. As a result 23 articles were identified and a further search at their references yielded 3 more. The contents of these articles were utilized in the preparation of the manuscript.

### Epidemiology

**Figure 1:**
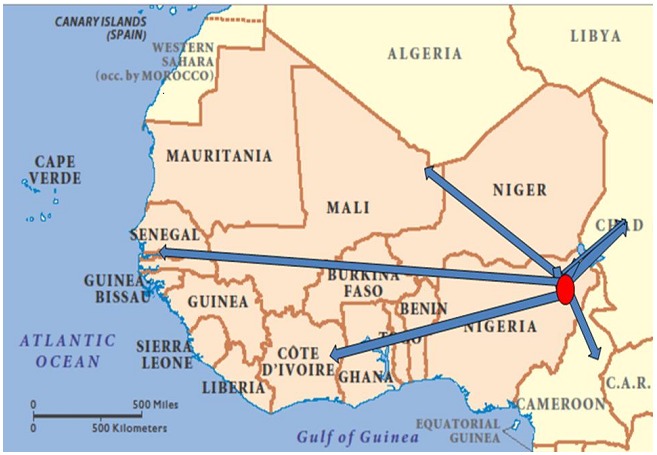
(Spread of Lassa fever from Lassa village Nigeria to West Africa and beyond Ibekwe; EFAS 2011). This schematic diagram depicts the spread of lass fever from West African sub region. The pink shaded areas are the hyper-endemic zones of the infection whereas the red spot and the arrows represent spread from Lassa village Nigeria.

Lassa fever is an acute hemorrhagic fever caused by Lassa virus(LAV), a bisegmented ambisense single-stranded RNA virus that belongs to the family old world *Arenaviridisae spp* [10]. It is prevalent in the West African sub-region where about 3–5 million [11] individuals are infected yearly. The first comprehensive identification of this illness was at Lassa village Nigeria in 1969, where 2 missionary nurses were infected and lost their lives in the process along with some other hospital workers [12,13]. Although there exists a report that this virus was first encountered in Sierra Leone (West Africa) about 10 years earlier [14]. Subsequently, the illness has spread within the sub-region and beyond (Figure 1). Cases have been reported in South Africa, Zambia and outside sub-Saharan Africa with devastating outcomes. The transmission is via the droppings of the multimammate rat (*Mastomys Natalensis Spp*) which serves as the reservoir to the virus. This rodent found around homes and farm settlements has some unique features which include long hairless tail, pointed rostrum and ventral surface lined by multiple [16–20] mammary glands(in females) from the thorax down to its abdomen [15]. On average it weighs 20–80g, the head to body measures about 6–17cm and the tail 6–15cm. This virulent rodent has an average life span of 2 years, breeds round the year with each pregnancy resulting in 16–20 litters [15].

Lassa fever (LF) is also nosocomial and transmitted through the body fluids of infected humans, fomites and aerosol. There is an associated high morbidity and mortality rates. It is speculated that at least 10,000 people die of Lassa fever in West Africa annually [16].

### Clinical features and sequeale of Lassa fever

The mode of presentation of Lassa fever can be non-specific and hence the difficulty in clinical diagnosis of some cases. However the classical modes of presentation include high grade fever >38°C, sore throat, retrosternal pain, cough, odynophagia, conjunctivitis, petechial hemorrhages, abdominal pains, vomiting and diarrohea. It could also present with headache, joint paint pains and facial swellings. The symptoms usually set in between 1^st^and 3^rd^ week of exposure to the LAV[17]. Throat examination usually reveals exudative pharyngitis and urinalysis often is characterized by proteinuria. The neutrophil count is often depressed. Neurological symptoms (tremors, convulsions, meningitis symptoms etc) are not commonly present at this early stage, however, sensorineural hearing loss sometimes presents. Recent research suggests that early sensorineural hearing loss and probably early manifestation of other CNS features depict poor prognostication [18,19]. The classical clinical course of Lassa fever is demonstrated in Table 1 [20].

**Table 1:** The classical clinical course of Lassa fever

Stage	Symptoms
1 (days 1–3)	General weakness and malaise. High fever, >39°C, constant with peaks of 40–41°C
2 (days 4–7)	Sore throat (with white exudative patches) very common; headache; back, chest, side, or abdominal pain; conjunctivitis; nausea and vomiting; diarrhoea; productive cough; proteinuria; low blood pressure (systolic <100 mm Hg); anaemia
3 (after 7 days)	Facial oedema; convulsions; mucosal bleeding (mouth, nose, eyes); internal bleeding; confusion or disorientation
4 (after 14 days)	Coma and death

The above data shows the usual pattern of the dramatic course of Lass fever disease within 2 weeks of patient becoming symptomatic. Source; [Reference 20] Merlin. ‘Licking' Lassa fever: a strategic review. London: Merlin, 2002 (http://www.merlin.org.uk/uploads/files/pr/Lassa%20Fever%20Strategy%202.pdf)

The morbidity and mortality is influenced by some key factors such as the time of presentation, diagnosis and commencement of appropriate treatment. Pregnancy carries a poor prognosis and most often fetuses are lost [21]. It is postulated that a single shot of appropriate dose of Ribavirin within the first week of symptom onset would reduce the mortality rate by 90% [12,19]. The case fatality rates vary among centers but could be as high as 16.5%–28% [22,23].

In a case study of 441 patients, McComick etal [22]observed that the best predictor of Lassa fever include the combination of fever, pharyngitis, retrosternal pain, and proteinuria (predictive value 0.81) whereas for the outcome, the best predictor was the combination of fever, sore throat, and vomiting (relative risk of death, 5.5). Furthermore, they observed the following complications: mucosal bleeding (17%), bilateral or unilateral eighth-nerve deafness (4%), and pleural (3%) or pericardial (2%) effusion. In a related study conducted among 908 patients by Inegbenebor et al in Nigeria; cultural practices and hygiene were identified as strong factors promoting the propagation of the disease [24].

Patients with subclinical infections to Lassa fever pass un-noticed. However, this group along with survivors of the acute Lassa fever illness stands the risk of developing sensorineural hearing loss of different degrees in future. The hearing loss is usually bilateral and can affect all frequencies of hearing [18] and according to WHO about 25% of patients exposed to LAV are affected [16]. The pathogenesis of this hearing loss is believed to follow an immunological reaction between the circulating Lassa virus antibodies and the basal cell membrane /outer hair cells of the cochlear [24].

Other neurological complications sometimes associated with survivors of Lassa fever are seizures, gait disturbances, tremors and encephalitis.

### Challenges in the management/control of Lassa fever

The high virulence and fatality rate of this disease is a major concern which is further complicated by the non-specific modes of presentation (mimicking some other fevers). From the foregoing, early presentation and subsequent diagnosis is usually not feasible especially in our hinterlands and villages. The contagious nature of the illness poses a big threat to the Medical attendants, other hospital workers and the care-givers who often are exposed to this disease unprotected, prior to diagnoses and establishment of barrier nursing. The fomite and aerosol mode of contraction posses a huge challenge to all who have close contact with the patients.

The control of the carrier vectors is herculean. The natural habitat of these rats within and around homes and farm settlements makes it cumbersome. Biological control by introduction of safe predators to rats like cats within the endemic areas tentatively carries some prospect; however the future consequences may be worse than the present. The ecosystem will totally be distorted because there is no way the cats will be able to selectively eliminate the Lassa rats alone. This line of reasoning could also be applied to why chemical control (rat pesticides) may also not suffice as an effective mode of control. Worse still, it may be possible that with time, following genetic transformations, such predators might transmute into Lassa Virus carriers. This will spell doom since these are household pets.

The inherent danger of complications that survivors and sub-clinically exposed individuals might suffer from is a concern. Studies had shown that survivors who developed severe sensorineural hearing losses had characteristic poor speech discrimination and were not amenable to hearing aids [18]. The role of antioxidants and hyperbaric oxygen [25,26] in ameliorating hearing losses and other neurological complications remains hypothetical.

LF and its lethal features qualify it as a suitable biological weapon. Although the aerosol mode of transmission posses threat to even the terrorist handling the virus, however it might be argued that giving the growing rate of suicide bombers, such handlers may be unperturbed about any inherent consequences. Furthermore, accidentally imported cases to the rest of the world had been reported with dire consequences making LF a global challenge and not just a problem confined to the developing world.

Unavailability of safe vaccines and cost effective/efficient rapid kit for diagnoses nearly half a decade after identifying the disease has hampered the containment of the illness. Obviously the future lies in effective and safe vaccination of the populace as in the case of yellow fever.

### Recommendations

At the grass root, a continuing education of the populace on the dangers of the disease, its modes of presentation and the need to seek medical treatment early should be intensified. Awareness (campaigns) and advocacy on clean and safe environment to promote prevention especially within the endemic areas are necessary. Abrogation of practices that might enhance contact with the LAV should be encouraged.

The secondary level of control should involve setting up serviceable diagnostic and treatment centers for Lassa fever within the West African sub-region and beyond to enhance prompt therapy and containment of the illness. The health personnel that work in such centers should be well protected, paid enhanced salaries and their lives insured. Well designed and funded researches should be instituted in this field to facilitate ameliorating breakthroughs.

Finally, the ultimate aim should be towards producing a functional and safe anti Lassa-fever-vaccine (which I refer to as tertiary control) that could be incorporated into the Immunization programs for children and adults respectively. The adverse immunological responses to Lassa antibodies such as hearing loss must be taken into cognition prior to the approval of such vaccine for human use.

## Conclusion

Prompt and effective containment of the Lassa fever disease in Lassa village and other mapped areas about five decades ago could have saved the West African sub-region and indeed the entire globe from the devastating effect and threats posed by this illness. There is still a need for more proactive measures towards the eradication of the illness at primary, secondary and tertiary levels of health care.
